# Dynamic antenna clustering along with low RF complexity digital beamforming

**DOI:** 10.1371/journal.pone.0325361

**Published:** 2025-06-26

**Authors:** Usman Haider, Syed Jawad Hussain, Imtiaz Ali Soomro, Sohaib Latif, Mrim M. Alnfiai

**Affiliations:** 1 Department of Computer Science, Sir Syed Case Institute of Technology, Islamabad, Pakistan; 2 Department of Computer Science and Software Engineering, Grand Asian University, Sialkot, Pakistan; 3 Department of Information Technology, College of Computers and Information Technology, Taif University, Taif, Saudi Arabia; Guangdong University of Petrochemical Technology, CHINA

## Abstract

This paper presents a digital beamforming technique with low radio frequency (RF) complexity that can preserve antenna weights between the antenna aperture and the digital baseband processing unit. Each antenna subarray connects a separate RF chain and it can only transmit one data stream. The RF chain is made to keep the beamforming weights of subarray antenna elements in a TDM fashion. In this paper, two dynamic clustering algorithms are proposed. Using the first algorithm, the actual number of required RF chains can be identified. By using the second algorithm, it is possible to comprehend the antennas in each subarray for a specific number of RF chains. RF circuit designers can utilize the proposal from this paper to determine the exact number of RF chains in a given scenario and the corresponding digital-to-analogue converter (DAC) and its associated components. Our findings show that, with a much lower RF complexity, the suggested digital beamforming scheme can successfully deliver almost the same spectral efficiency as a full-complexity DBF system.

## 1 Introduction

The radiation pattern of a single antenna is fixed, but an array of antennas has the ability to modify it over frequency and time. Antenna arrays utilizing beamforming can mitigate the losses associated with high frequency (mmWave 30–300 GHz) and propagation attenuation by enhancing the gain at both the transmitter and receiver, thereby forming directive beams [1, 2]. Massive Multiple Input Multiple Output (MIMO) antenna arrays enable concentration of transmitted power in specific directions and the formation of multiple narrow beams, which improves spatial filtering, signal-to-noise ratio (SNR), and spectral efficiency [3]. The directivity of the beams relies heavily on precise control over the amplitude and phase of the transmitted signal at each antenna. Analogue, hybrid, and fully digital beamforming techniques have been integral to antenna transmission for decades and are used alternately across different wireless communication systems. The core aspect of these techniques is how information is transferred and preserved from the baseband to the antenna aperture and vice versa in the receive scenario.

For instance, in analogue beamforming, a single RF chain can be connected to $n_{\text{T}}$ antennas using analogue phase shifters. The signal received from these $n_{\text{T}}$ antennas is summed in the analogue domain after weighting, which reduces the signal dimensionality from $n_{\text{T}}$ to 1. As a result, the amplitude and phase information of each antenna cannot be independently retrieved at baseband, limiting the effectiveness of transmit precoding. Hybrid beamforming employs multiple analogue phase arrays to handle phase shifting in the RF/IF domain, while digital baseband processing is used for the remaining operations [4]. The analogue sub-arrays (consisting of *n*_T_ antennas) are connected to *n*_RF_ RF chains for spatial multiplexing. However, spatial resolution—necessary for generating closely spaced transmitted beams—is constrained due to the lack of independent control over each antenna element. Digital beamforming (DBF) is optimal by definition, as it preserves the amplitude and phase weighting of the input signal from the digital baseband processing unit to the antenna aperture [5]. This allows DBF to achieve accurate beam steering, precise pattern shaping, and the generation of multiple closely spaced beams, while also eliminating interference by directing nulls towards interferers [5–8]. Typically, a separate RF chain is used for each antenna in a DBF system to maintain the signal weighting performed by the digital processing unit. However, such an implementation becomes challenging for large-scale antenna systems [9]. The primary drawbacks of full-complexity DBF include high costs, increased power consumption, and a significantly larger hardware form factor due to the higher number of RF chains.

### 1.1 Contribution

In this paper, we introduce a fully dynamic low RF complexity DBF scheme that does not require the number of RF chains to be equal to the number of antennas.

- Our proposal forms antenna clusters, and each antenna cluster requires one RF chain per cluster.
- Our proposal has the ability to selectively use the available RF chains by forming the dynamic antenna clusters.
- Our proposal can be used to estimate the actual number required RF chains and find the actual antennas in each cluster.

Simulation results demonstrate that our approach achieves channel capacity comparable to full RF complexity DBF while significantly reducing RF chains. The rest of the paper is organized as follows: Sect 2 presents the system model and dynamic antenna grouping. Sect 3 provides a brief description of the MIMO channel model. Sect 3.2 discusses the dynamic clustering of subarrays for the proposed low RF complexity digital beamforming. Sect 4 presents the simulation results for both algorithms, and Sect 5 concludes with future work.

## 2 Literature review

Over the past decade, hybrid beamforming techniques combining digital processing with analog beamforming have been widely explored as an alternative to fully digital beamforming (DBF) due to their reduced RF complexity, although at the cost of lower robustness and suboptimal performance [4, 10]. However, in an era dominated by digital advancements, the authors of [11] raise an intriguing question: “Why is cellular technology seemingly shifting away from digital approaches and reverting to analog-based solutions?" This perspective challenges the current trend in beamforming design and highlights the ongoing debate about the optimal balance between digital and analog processing in modern wireless systems.

Contrary to conventional belief, prior research [12–15] has demonstrated that digital beamforming (DBF) can be achieved even with a single RF chain while maintaining performance comparable to traditional DBF systems with multiple RF chains. The primary goal of DBF is to ensure signal granularity and retain the digital domain signal until it reaches the antenna array. To achieve this, low-complexity systems employ multiplexing techniques, commonly used in cellular networks [16], to preserve digitally processed signals across multiple antenna elements through a shared RF chain. One such approach, the multi-tone weighting scheme introduced in [14], assigns unique frequency tones to each antenna signal and combines them for joint processing. This method is similar to frequency-division multiple-access (FDMA) in wireless communication, with the RF chain serving as a shared processing channel rather than a wireless transmission channel. Despite its advantages, this approach has notable drawbacks. It requires independent frequency tone generators for each antenna during both multiplexing and demultiplexing. Additionally, nonlinearity in analog components, phase noise in oscillators, gain imbalances, and signal leakages within local oscillators (LO) and mixers can lead to signal distortion [17]. While in-phase and quadrature imbalance calibration techniques [18, 19] can mitigate some of these issues, implementing them in large antenna arrays with a multi-tone weighting approach increases RF complexity, as each antenna requires separate calibration [18]. The recent work of FDM based RF complexity reduction presented in [20] shows the application of low RF complexity DBF systems. The proposal in [20] used a realistic prototype of 4 antenna elements connected with one RF chain having a single ADC. The RF complexity reduction is done by reducing the bandwidth of each antenna instead of increasing the overall sampling rate of ADC. Therefore, the reduction of overall bandwidth by a factor of $\frac{B}{N}$ results a reduction in the overall data rate or alternatively increasing bandwidth requires an ADC with significantly higher sampling rate and it may increase the overall cost and power consumption of the circuit.

An alternative solution, the time-sequence phase weighting (TSPW) technique proposed in [12, 21], applies digital phase weighting by post-multiplying signals with a Walsh-Hadamard matrix, introducing controlled phase shifts and enabling multiplexing over a single RF chain. At the antenna array, a phase-shifting network is designed to act as an inverse Walsh-Hadamard transform, allowing signal recovery. While effective for narrowband signals, this technique faces performance degradation when applied to wideband signals, especially in large-scale antenna arrays, as observed in [22, 23]. The challenges associated with wideband implementations become more significant as the array size increases, further emphasized in [24].

A time-switching phased waveform (TSPW) approach was explored in [21] for its potential in anti-jamming radar applications. Building upon this, the study in [13] introduced compressed sensing techniques into the TSPW framework to lower the digital-to-analog converter (DAC) sampling rate by deactivating certain antenna elements. This, however, comes with a trade-off in beamforming performance. One of the key advantages of TSPW-based systems is the minimal hardware requirement, relying on only a single DAC and a local oscillator (LO). Despite this simplicity, achieving performance comparable to conventional digital beamforming (DBF) in large antenna arrays necessitates a DAC with a sampling rate of $f_{s}=N\;\times \;B$, where *N* is the number of transmitting antennas and *B* is the system bandwidth. Furthermore, to reverse the Walsh-Hadamard transform applied in the digital domain, a complex analog phase shifter network is required at the RF front end.

The work in [15] introduces a time-division multiplexing (TDM) approach to optimize RF resource utilization, aiming to minimize RF complexity. The study in [15] introduced the Spatial-MultIplexing-of-Local-Elements (SMILE) architecture, which utilizes time-multiplexing of baseband signals across a shared RF chain. The SMILE architecture is designed to operate at a sampling frequency $f_{s}\geq N\;\times \;B$, where *N* denotes the number of antenna elements and *B* is the system bandwidth. The multiplexed signal is reconstructed in the RF domain through demultiplexing and band-pass filtering. Apart from its hardware simplicity, SMILE can achieve a signal-to-noise ratio (SNR) performance comparable to that of fully digital beamforming (DBF) architectures with complete RF chains. To address distortion introduced by inter-channel interference in time-multiplexed signals, a compensation mechanism was proposed in [25]. Furthermore, the work in [26] implemented a SMILE-based system using orthogonal beams for signal encoding, enabling digital beamforming via a single RF receiver. A hardware prototype featuring four antennas and a single DAC was presented in [27], demonstrating the practical feasibility of the concept.

Additional challenges associated with SMILE, such as reduced radiation efficiency and mismatches in sampling rates, were addressed in [28] and [29], respectively. Despite these advancements, implementing SMILE in large-scale millimeter-wave (mmWave) systems remains challenging due to the stringent requirement that both DAC sampling rate and antenna switching speed meet or exceed $f_{s}\geq N\;\times \;B$. This limitation hampers scalability. To alleviate this constraint, [30] proposed a solution that relaxes the high-speed requirements at the cost of reduced system bandwidth, thereby leading to a trade-off in overall system capacity.

The analysis of contemporary DBF techniques highlights the challenges associated with their implementation in mmWave systems featuring extensive antenna arrays. As a result, hybrid and analog beamforming methods, despite being sub-optimal and less robust, have gained widespread acceptance in mmWave research. Nevertheless, a pivotal study in [31] critically evaluates the implementation challenges and performance trade-offs of these techniques. Findings from [8, 31] suggest that DBF remains the most viable approach for realizing 5G and beyond mmWave wireless networks. This insight serves as the primary motivation for our research, where we employ an antenna clustering strategy, assigning one RF chain per antenna cluster to enhance system efficiency.

The concept of subarray formation has been explored extensively in prior research. Existing approaches, such as those in [32, 33], are primarily applicable to full RF complexity systems and small uniform linear antenna arrays. Similarly, dynamic subarray selection methods have been proposed in hybrid precoding studies [34], where predefined codebooks guide the selection process. Our approach differs by introducing a DBF technique that significantly reduces RF complexity while achieving spectral efficiency and robustness comparable to a full RF complexity DBF system. Unlike the fixed subarray structures discussed in [8], where antenna grouping remain static, our method introduces a fully dynamic subarray structure. This allows for adaptable RF chain utilization, activating only the necessary RF chains based on real-time requirements. The proposed DBF technique leverages dynamic subarray formation combined with time-division multiplexing (TDM) to maintain weight preservation from the baseband to the antenna aperture. The motivation for this dynamic subarray approach is supported by Eq 1, which indicates that, within a system of a given bandwidth, reducing the number of active antennas lowers the required sampling rate and switching speed for TDM-based beamforming.

## 3 System model

**Fig 1:**
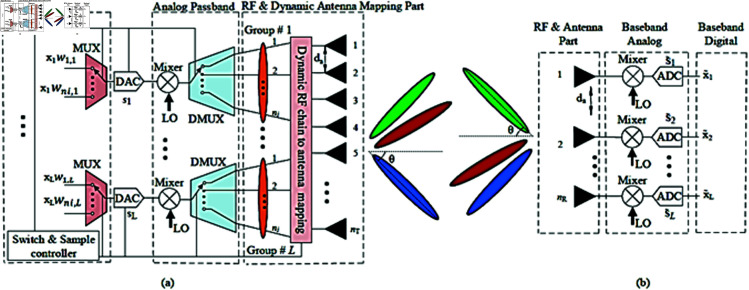
(a) Proposed dynamic low RF-complexity DBF architecture. (b) Conventional full RF-complexity DBF at the receive side.

Consider a single-user downlink MIMO system with $n_{\text{T}}$ transmit antennas and $n_{\text{R}}$ receive antennas, where $n_{\text{T}}\gg n_{\text{R}}$. The system is designed with fewer RF chains than transmit antennas, i.e., $n_{\text{RF}}<n_{\text{T}}$, and each RF chain is dynamically mapped to a subarray within the transmit antenna array, as illustrated in Fig 1. The digitally processed baseband signal *s*_*i*_, corresponding to each antenna element within the subarray, is transmitted through the RF chain using time-division multiplexing (TDM). In this configuration, the sampling rate required for transmit beamforming must exceed the product of the number of active antenna elements $n_{i}\;\left(\sum_{\forall i}n_{i}=n_{\text{T}}\right)$ within a subarray and the signal bandwidth *B*, leading to a minimum requirement of $B\;\times \;n_{i}$ [15]. Additionally, the switching speed of the RF chain to the antenna mapping network must meet this same requirement for each subarray. The subarray formation is dynamically adjusted based on the instantaneous spatial correlation of the channel. Consequently, for each channel realization, different sets of antennas may be selected, leading to variations in *n*_*i*_ across snapshots. This variation directly impacts the minimum switching speed needed for mapping the RF chain to the antenna elements. Assuming a common clock regulates the sampling rate of the signal *s*_*i*_ across all RF chains, the resulting sampling rate is given by:

$$f_{s}\geq B\;\times \;\max(n_{1},n_{2},\cdots ,n_{G_{t}})$$ (1)

Let *x*_*i*_ be the input signal weighted by a coefficient *w*_*i*_ in the digital processing unit, then the preserved TDM signal $s_{i}$ at the antenna aperture of $i^{\text{th}}$ subarray is defined as

$$\begin{array}{c} s_{i}\left(t\right)=\sum_{m=1}^{n_{i}}w_{m,i}x_{i}\delta (t-mt_{s}) \end{array}$$ (2)

where Dirac delta (δ) represents an impulse train propagating through a single RF channel (chain) and $t_{s}=\frac{1}{f_{s}}$ is the sampling time of digitally pre-processed symbols. System model of our proposed low RF complexity DBF technique and full RF complexity DBF are shown in Fig 1.

Let $H\in C^{n_{\text{R}}\;\times \;n_{\text{T}}}$ be a single user downlink MIMO channel matrix which is perfectly known at the transmitter, $s\in C^{n_{\text{T}}\;\times \;1}$ be the digitally precoded transmit signal vector preserved through the RF chain, then the receive signal vector $y\in C^{n_{\text{R}}\;\times \;1}$ can be written as

$$\begin{array}{ccc} y & = & Hs+z\\ & = & \sum_{i=1}^{G_{\text{T}}}H_{i}w_{i}x_{i}+z \end{array}$$ (3)

where, $H_{i}\in C^{n_{\text{R}}\;\times \;n_{\text{i}}}$ is the subarray channel matrix selected from H, $w_{i}\in C^{n_{i}\;\times \;1}$ weight vector from the digital processing unit and z is the additive white Gaussian noise (AWGN) process with zero mean and unit variance and it has a circularly symmetric probability density function (PDF).

### 3.1 Channel model

Assume that a multipath propagation channel consists of *N*_*cl*_ distinct multipath clusters, each characterized by *N*_*ray*_ intra-cluster multipath components (MPCs). Let the azimuth angle of arrival and departure of the $l^{\text{th}}$ ray within the $i^{\text{th}}$ cluster be denoted by $\theta_{i,l}^{\text{A}}$ and $\theta_{i,l}^{\text{D}}$, respectively. In a similar manner, $\phi_{i,l}^{\text{A}}$ and $\phi_{i,l}^{\text{D}}$ represent the elevation angles of arrival and departure for the corresponding ray. The MIMO channel matrix $H(\tau )\in {\mathbb{C}}^{n_{\text{R}}\;\times \;n_{\text{T}}}$ can then be expressed as:

$$\begin{array}{ccc} H\left(\tau \right) & = & \sum_{i=1}^{N_{cl}}\sum_{l=1}^{N_{ray}}\alpha_{i,l}\sqrt{L\left(r_{i,l}\right)}a_{\text{R}}\left(\phi_{i,l}^{A},\theta_{i,l}^{A}\right)\;\times \;\\ & & a_{\text{T}}^{H}\left(\phi_{i,l}^{D},\theta_{i,l}^{D}\right)h\left(\tau -\tau_{i,l}\right)+H_{\text{LOS}}\left(\tau \right) \end{array}$$ (4)

where the channel impulse response matrix of the LOS component arriving at delay τ is denoted as $H_{\text{LOS}}(\tau )$. The transmit and receive antenna array steering vectors are represented by $a_{\text{T}}$ and $a_{\text{R}}$, respectively. The impulse response at the delay tap τi,l of the $l^{\text{th}}$ ray within the $i^{\text{th}}$ cluster is given by $h(\tau -\tau_{i,l})$. Additionally, the complex channel gain and attenuation loss of the $l^{\text{th}}$ ray in the $i^{\text{th}}$ cluster are denoted by $\alpha_{i,l}$ and *L*(*r*_*i*,*l*_), respectively. Since the LOS component has the most energy, blocking modelling of this component is significantly interesting. Therefore, the line-of-sight component of the channel model is given as

$$\begin{array}{ccc} H_{\text{LOS}}\left(\tau \right) & = & I_{\text{LOS}}\left(d\right)\sqrt{n_{\text{R}}n_{\text{T}}}e^{j\eta }\sqrt{L\left(d\right)}\;\times \;\\ & & a_{\text{R}}\left(\phi_{i,l}^{A},\theta_{i,l}^{A}\right)a_{\text{T}}^{H}\left(\phi_{i,l}^{D},\theta_{i,l}^{D}\right)h\left(\tau -\tau_{i,l}\right) \end{array}$$ (5)

where, η~𝒰(0,2π) denotes the line-of-sight component’s phase and $I_{\text{LOS}}\left(d\right)$ presents a random-variable that indicates whether a line-of-sight link exists at a *d* distance among the transmit and receive sides. We employed the same measurement-based approach described in [35] to determine the likelihood *p* of LOS connection blockage. In case of a typical street-canyon scenario, the probability of line-of-sight is given as

$$p=\min\left(\frac{20}{d},1\right)\left(1-e^{-\frac{d}{39}}\right)+e^{-\frac{d}{39}}$$ (6)

whereas, for a shopping mall scenario, it is defined by

$$p=\left\{ \begin{array}{cc} 1 & d\leq 1.2\\ e^{-(\frac{d-1.2}{4.7})} & 1.2<d\leq 6.5\\ 0.32e^{-(\frac{d-1.2}{4.7})} & d\geq 6.5 \end{array}\right.$$ (7)

Let *n*_*f*_ correspond to the number of frequency bins, then for our simulations, channel matrix H is normalized such that

$$\sum_{i=1}^{n_{\text{R}}}\sum_{j=1}^{n_{\text{T}}}\sum_{f=1}^{n_{f}}|H\left(i,j,f\right){|}^{2}=1\cdot$$ (8)

**Fig 2:**
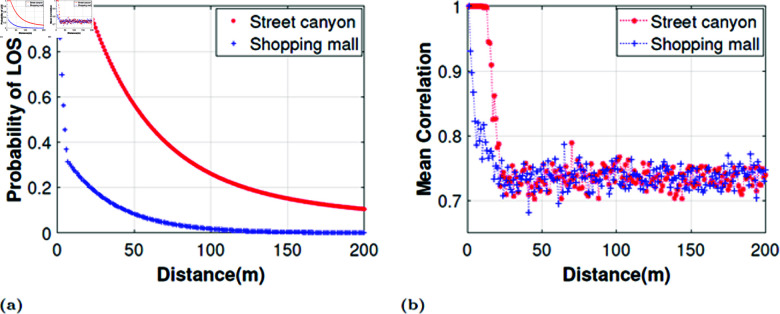
Probability of LOS models and average transmit correlation analysis for 64×32 MIMO system in street canyon and shopping mall scenarios, (a) Probability of LOS models. (b) Mean transmit correlation.

**Fig 3:**
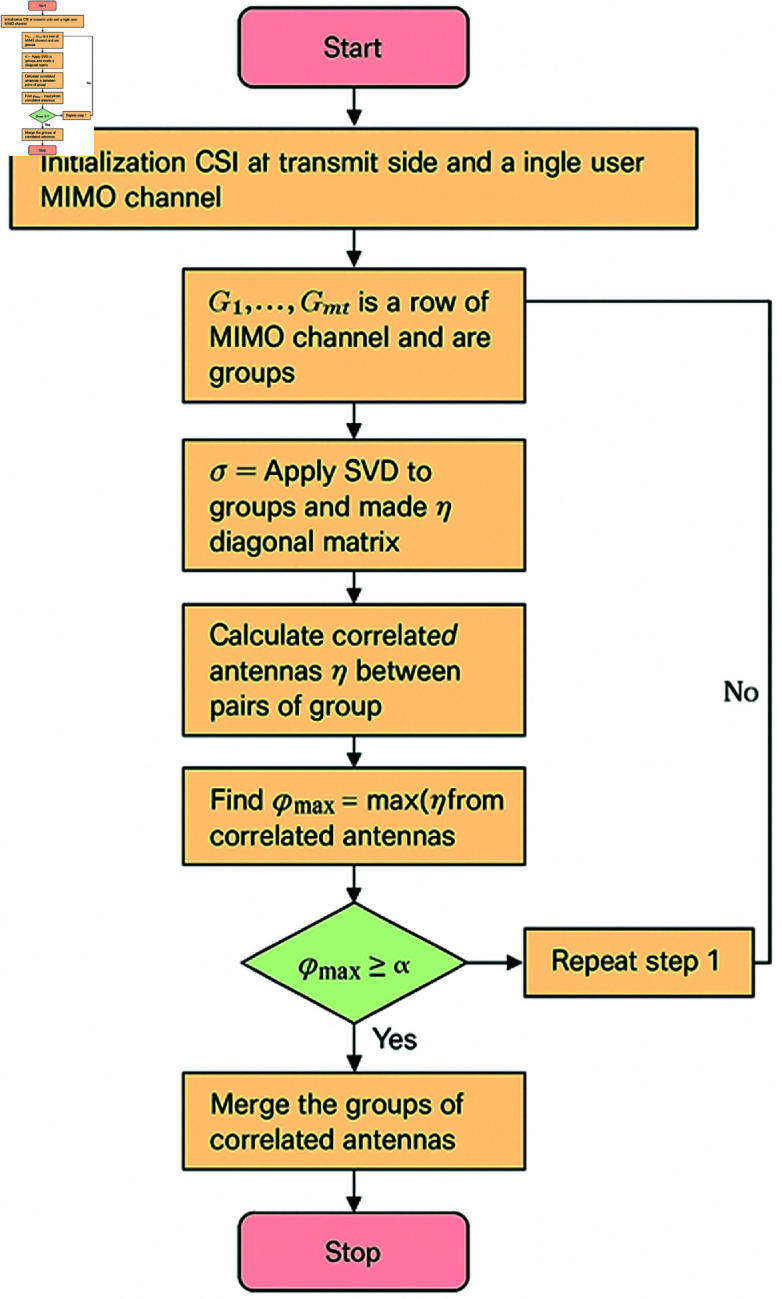
Flowchart of the study.

Fig 2 shows the probability of LOS models defined in Eqs 6 and (7) and the scaling of average transmit correlation with the probability of LOS. One can see that the mean correlation follows the trend of the probability of LOS. These results are quite intuitive and resemble the realistic measurement results shown in [36, see Fig. 3] greatly. This validates the theoretical accuracy of our channel model setup. It is interesting to note that, a radio channel (including antenna as a part of the channel) is composed of both high and low-correlated antenna pairs or groups. This property of the channel can be effectively used together with singular value decomposition (SVD) based beamforming. Notice that, SVD-based beamforming benefits greatly from high spatial correlation in such a way that it results in increasing the strength of dominant eigenmode, which in turn results in close to rank-1 channels. Consequently, the remaining eigenmodes lose their significance for being a transmission candidate. Therefore, our proposed DBF intends to divide the whole transmit array into subarrays such that each subarray forms a close to rank-1 subchannel with receive antenna elements. Some other selected statistical features and model distributions are summarized in the following section, whereby a detailed description is available in [37]. The study considers a shopping mall or street canyon environment with a carrier frequency of 73 GHz. The number of clusters (*N*_*cl*_) is fixed at 10, while the number of rays per cluster follows a uniform distribution in the range [1,30]. The azimuth angle of arrival (AOA) and azimuth angle of departure (AOD) per cluster follow a Laplacian distribution, with the mean uniformly distributed in [0,2π] and $\left[-\frac{\pi }{2},\frac{\pi }{2}\right]$, respectively, and a standard deviation of $5^{\circ }$. Similarly, the elevation angle of arrival (AOA) and elevation angle of departure (AOD) per cluster also follow a Laplacian distribution, with the mean uniformly distributed in $\left[-\frac{\pi }{2},\frac{\pi }{2}\right]$ and a standard deviation of $5^{\circ }$. The system employs transmit antenna arrays configured as either (8×8) Uniform Planar Arrays (UPA), corresponding to 64 antennas, while the number of receiver is equipped with 32 antennas. The antenna spacing is set at 0.5λ, ensuring optimal performance. The transmit and receive antennas are positioned at heights of 7 meters and 1.68 meters, respectively, with an average transmit-receive distance of 200 meters. Both the transmit and receive antenna orientations are arbitrary, allowing flexibility in deployment scenarios.

The work in [38] analyzed two array configurations—Polarized Uniform Linear Array (PULA) and Stacked Polarized Uniform Circular Array (SPUCA)—and demonstrated that line-of-sight (LOS) propagation scenarios exhibit higher spatial correlation compared to non-line-of-sight (NLOS) conditions. As a result, increased spatial correlation negatively impacts both channel capacity and diversity gain.

In the context of millimeter-wave (mmWave) multiple-input multiple-output (MIMO) systems, which typically employ large antenna arrays with densely spaced elements, elevated spatial correlation is anticipated. Motivated by this, we conducted a statistical investigation of spatial correlation in mmWave MIMO channels. Therefore, we used the extreme cases where the α is assumed very close to 1. It can be noted that when α→1, each antenna in a group becomes fully correlated to all the antennas in the group. For modeling the probability of LOS links, we adopted the approach outlined in [35, 39].

### 3.2 Dynamic subaray formation for proposed low RF complexity DBF

For simplicity of the system design, we assume equal power allocation to each antenna element of the transmit array. Thus channel capacity of full complexity DBF can be defined as

$$C_{\text{FC}}=\log\det\left(I_{n_{\text{R}}\;\times \;n_{\text{R}}}+\frac{\text{SNR}}{n_{\text{T}}}H^{H}H\right)\cdot$$ (9)

Subarray formation is an important part of the proposed DBF, because it primarily aims to reduce the sampling rate of the TDM signal in the RF chain which in turn results in reduction of the switching speed of the RF chain to antenna mapper shown in Fig 1. The sum capacity of channels decomposed into subarrays is defined by Telatar in [41] as

$$C_{\text{SA}}=\log\det\left(I_{n_{\text{R}}\;\times \;n_{\text{R}}}+\frac{\text{SNR}}{n_{\text{T}}}\sum_{i=1}^{G_{\text{T}}}H_{i}w_{i}w_{i}^{H}H_{i}^{H}\right)\cdot$$ (10)

Theoretically, Eq 10 converges to Eq 9 as $G_{\text{T}}\to n_{\text{T}}$. Since in our proposed DBF, we assume that $G_{\text{T}}=n_{\text{RF}}$, then for a particular $n_{\text{RF}}$, maximization of Eq 10 requires maximizing $H_{i}w_{i}w_{i}^{H}H_{i}^{H}$. This requires selection of close to rank-1 subchannels $H_{i}$ from the full MIMO channel H. We assume that $w_{i}$ is weight vector corresponding to the dominant singular value of $H_{i}$. Since fading is a random process, therefore, one cannot expect the propagation environment to result in a deterministic spatial correlation profile at the aperture of multi-antenna element array. Consequently, the selection of $H_{i}$ with an equal number of antenna elements per subarray is intuitively suboptimal. This motivates us to select subarrays based on the instantaneous channel state information (CSI) with a variable number of antennas per subarray. Let *r* be the rank and $\sigma_{1}\geq \sigma_{2}\geq \cdots \geq \sigma_{\text{r}}$ be the singular values of $H_{i}$, then closeness of *r* to 1 can be measured as

$$\begin{array}{c} \eta =\frac{\sigma_{1}^{2}}{\sum_{i=1}^{r}\sigma_{\text{i}}^{2}} \end{array}$$ (11)

where, 0≤η≤1. The convergence η→0 explains that all columns of $H_{i}$ belong to mutually orthogonal subspaces. In contrast, η→1 demonstrates that all columns of $H_{i}$ are linearly dependent and highly correlated. Consequently, $H_{i}$ becomes a rank-1 channel.

In general, a MIMO system is always equipped with a fixed number of RF chains ($n_{\text{RF}}$). The value of $n_{\text{RF}}$ is of fundamental interest to a system designer. Eq 10, The high-level implementation of the proposed algorithm is given as

## 4 Simulation results

**Fig 4:**
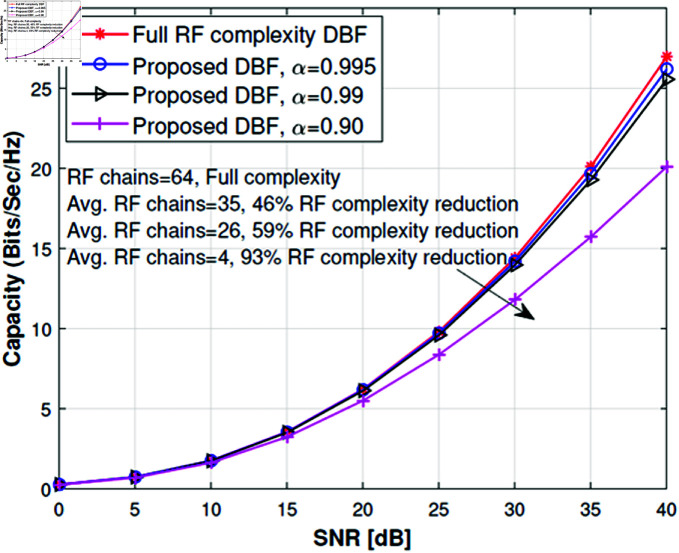
Channel capacity of 64×32 MIMO system with variable α.

We have considered a 64×32 MIMO downlink scenario with 64 transmit antennas employed at base station and a receiver equipped with 32 receive antennas. The simulation is set up for LOS and NLOS scenarios and details of other system parameters are given in Table 1. We have selected the RF complexity and MIMO channel capacity as a performance metric to evaluate the performance of the proposed low RF complexity DBF and compared it with full complexity DBF. For this purpose, we have formed antenna subarrays using Algorithm 1 and selecting the α=0.995,0.99 and 0.90. Selecting such high values of α enables us to make the cutoff threshold very tight for η. This helps form subarrays of the antenna such that the rank of the associated channel is very close to 1. Using such configuration will help us to understand how many RF chains are required if we want to achieve the same channel capacity equivalent to full complexity DBF. It can be observed from Fig 4 that the channel capacity of proposed low RF complexity DBF is very close to full complexity DBF for α=0.995 because the effective rank of the channel of each subarray becomes almost 1 when η tends to 1.

**Algorithm 1. Dynamic antenna cluster formation for Digital transmit beamforming with a fixed number of antenna groups.**

1: **Initialization :** CSI known at transmit side

2: For $H\in C^{n_{\text{T}}\;\times \;n_{\text{R}}}$ Consider every row a group

3:    ${\text{G}}_{1}\to {\text{H}}_{1}=\left[{\text{h}}_{11}\cdots {\text{h}}_{1n_{\text{R}}}\right]\in C^{1\;\times \;n_{\text{R}}}$

4:    :

5:    $\text{G}{\}}_{n_{\text{T}}}\to {\text{H}}_{n_{\text{T}}}=\left[{\text{h}}_{n_{\text{T}}1}\cdots {\text{h}}_{n_{\text{T}}n_{\text{R}}}\right]\in C^{1\;\times \;n_{\text{R}}}$

6: **Step 1:**

7:    $\sigma =svd\left[\begin{array}{c} {\text{G}}_{\text{i}}\\ {\text{G}}_{\text{j}} \end{array}\right]$ ⇒ $diag\left(\sigma \right)=\sigma_{1},\sigma_{2},\cdots ,\sigma_{\text{r}}$

8: where

9:    $\sigma_{1}>\sigma_{2}>\cdots >\sigma_{\text{r}}$

10: **Step 2:**

11: **Calculate** **η** **between every pairs of groups**

12:    $\eta_{\text{i},\text{j}}=\frac{\sigma_{1}^{2}}{\sum_{i=1}^{r}\sigma_{\text{i}}^{2}}$

13: **Step 3:**

14:    **Find** $\Phi =\max\left(\eta_{\text{i},\text{j}}\right)$

15: Merge the groups ${\text{G}}_{\text{i}}$ and ${\text{G}}_{\text{j}}$

16:    ${\text{G}}_{\text{m}}=\left[\begin{array}{c} {\text{G}}_{\text{i}}\\ {\text{G}}_{\text{j}} \end{array}\right]\in C^{\text{m}\;\times \;n_{\text{R}}}$

17: ${\text{G}}_{\text{m}}$rows>=Fixed Number of RF chains

18: **Repeat Steps 1 and 2 until required groups remain**

**Table 1:** A chart of associated parameters, along with various other miscellaneous parameters [39].

Parameter	Distribution	Mean & St. dev.
Azimuth angle-of-arrival per cluster *i*	Laplacian	$\theta_{i}^{A}\sim 𝒰[0,2\pi ]$ & $5^{\circ }$
Azimuth angle-of-departure per cluster *i*	Laplacian	$\theta_{i}^{D}\sim 𝒰[\frac{-\pi }{2},\frac{\pi }{2}]$ & $5^{\circ }$
Elevation angle-of-arrival per cluster *i*	Laplacian	$\phi_{i}^{A}\sim 𝒰[\frac{-\pi }{2},\frac{\pi }{2}]$ & $5^{\circ }$
Elevation angle-of-departure per cluster *i*	Laplacian	$\phi_{i}^{D}\sim 𝒰[\frac{-\pi }{2},\frac{\pi }{2}]$ & $5^{\circ }$
Total-Clusters $\left(N_{cl}\right)$	10	
Number of rays-per-cluster	Uniform [1,30] [40]	
Single user MIMO chanel	H	
Additive white Gaussian noise	z	
Number of frequency bins	*n*_*f*_	
Diagnol Matrix	σ	
Co related antennas	η	
Max Co-related	Φ	

In this case, the dominant eigenmode will be stronger while the other eigenmodes will lose their significance; discarding them will not show a noticeable loss in channel capacity. The strict criterion of α causes more groups to add multiplexing gain. It can be noted that the proposed low RF complexity DBF can achieve a comparable performance with 46% average reduction in RF complexity. The remarkable results for α=0.99 and 0.90 shows an average reduction in RF complexity up to 59% and 93% respectively, with minor degradation in channel capacity. The main reason for the loss in channel capacity is that the number of antennas per group increases when we relax α and the effective rank of the channel matrix increases. In this case, dominant eigenmode transmission per subarray will lose the channel capacity because of discarding other eigenmodes which could have contributed to channel capacity. When comparing the RF complexity, the required RF chain changes from 1 to 64 in Fig 7a based upon the channel spatial correlation properties. There is a major RF complexity reduction in the case of α=0.90. Although the RF complexity can be reduced by lowering the α threshold but the number of antennae per subarray increases as shown in Fig 7b and it will require high-speed antenna switching. We intend to reduce RF complexity by forming antenna subarrays in a TDM way such that the antenna switching speed must not be very high; which means subarrays with a small number of antennas can be easily realized in practical systems. Therefore a careful design is required to reduce RF complexity with the constraint of switching speed.

**Fig 5:**
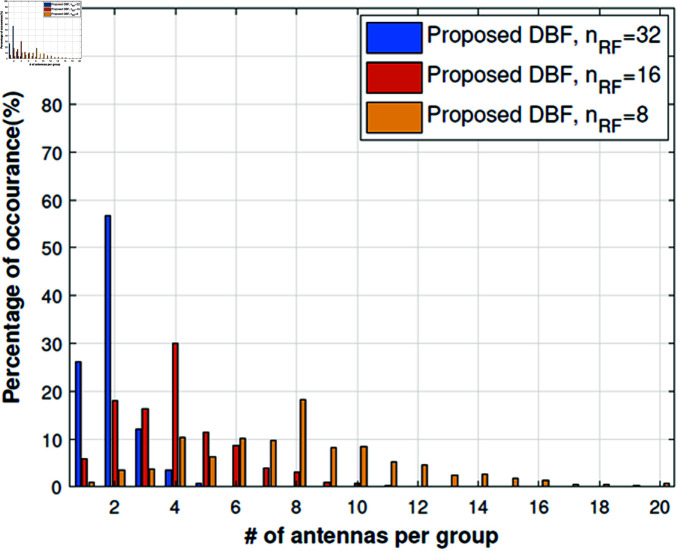
Number of selected antennas per group, LOS street canyon scenario.

**Fig 6:**
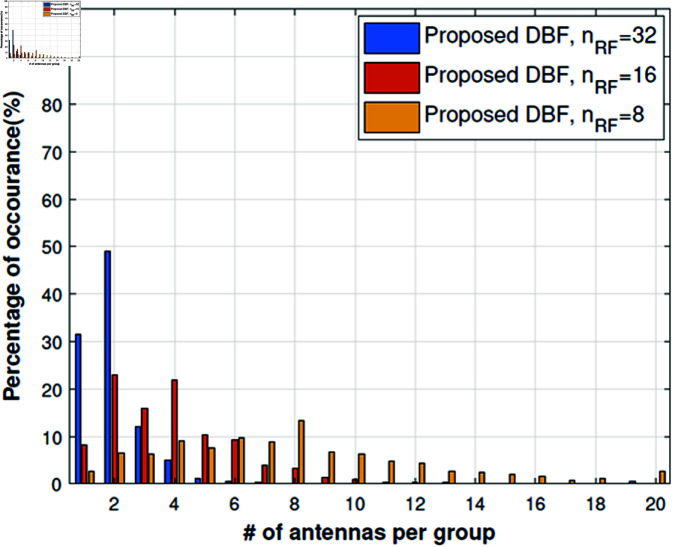
Number of selected antennas per group, NLOS street canyon scenario.

We have extended the study to understand actual antenna switching requirements by using Algorithm 2 to check statistically how many antennas are required per subarray. For this purpose, we have kept the RF chains fixed i.e., *n*_*RF*_ = 32,16,8. The results in Figs 5, 6 show that if we use 32 RF chains then mostly the number of antennas per subarray is up to 4 and it can be implemented easily as proposed in [15]. The simulation results are very convincing too because channel capacity remains very close to full complexity DBF. When we reduce RF chains (*n*_*RF*_ = 16,8), the channel capacity decreases slightly with a significant reduction in RF complexity as shown in Figs 8, 9. Although the number of antennas per group increases, which will require higher antenna switching speed, *n*_*RF*_ = 16,8 setups give more diversity and beamforming gain as compared to *n*_*RF*_ = 32 setup. A slight loss in channel capacity can be noted from Fig 8 for the LOS scenario as compared to NLOS given in Fig 9. The main reason for the decrease in channel capacity is overall highly correlated antenna pairs increase and the number of antennas per group increases for the LOS scenario in Fig 5. Our proposed algorithm can achieve the channel capacity near to full complexity DBF with 32 RF chains, which is a straightforward 50% reduction in RF complexity. Overall the channel capacity results are quite promising for 16 and 8 RF chains.

**Fig 7:**
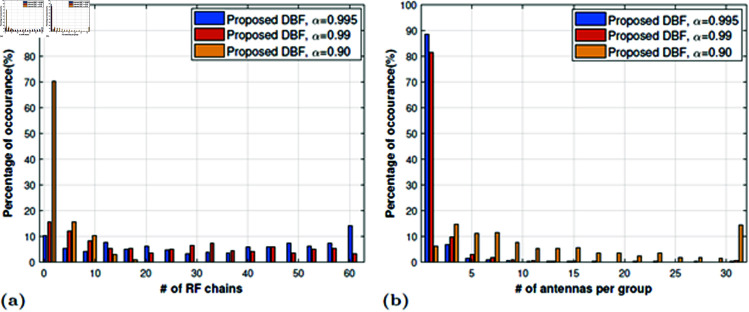
Statistical analysis of the (a) Percentage of several subarrays formed (same as the required number of RF chains). (b) Percentage of number of antennae ni per subarray. 64×32 MIMO system.

**Fig 8:**
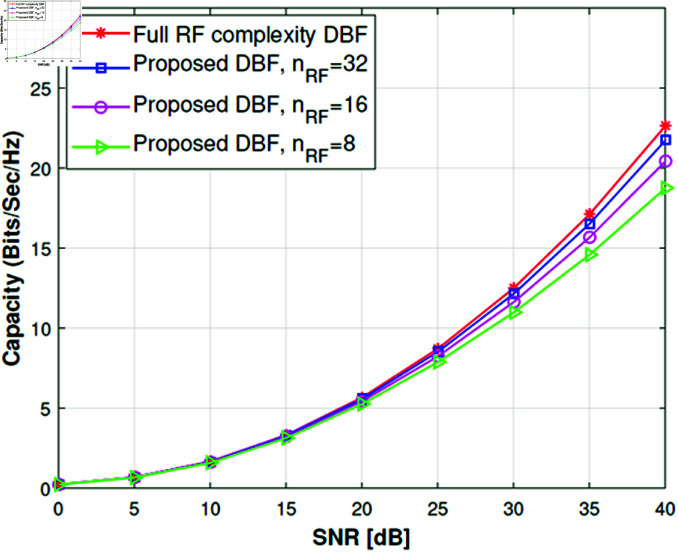
Channel capacity of 64×32 MIMO system with LOS street canyon scenario.

**Fig 9:**
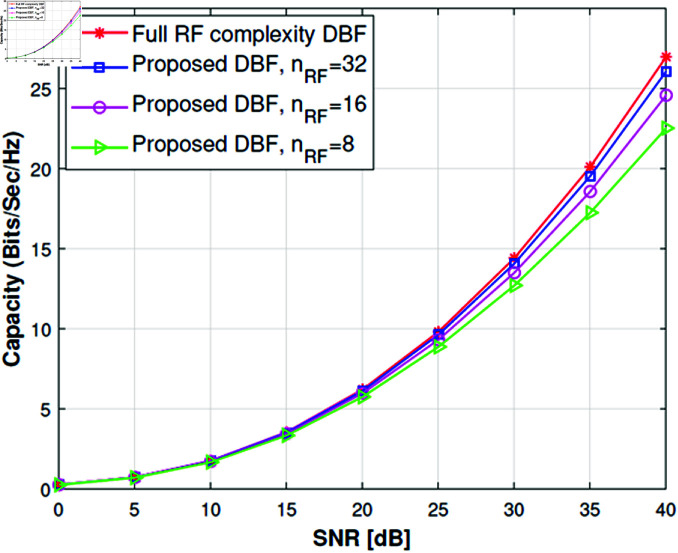
Channel capacity of 64×32 MIMO system with NLOS street canyon scenario.

**Algorithm 2. Dynamic antenna cluster formation for Digital transmit beamforming based on rank-1 threshold.**

1: **Initialization :** CSI known at transmit side

2: For $H\in C^{n_{\text{T}}\;\times \;n_{\text{R}}}$ Consider every row a group

3:    ${\text{G}}_{1}\to {\text{H}}_{1}=\left[{\text{h}}_{11}\cdots {\text{h}}_{1n_{\text{R}}}\right]\in C^{1\;\times \;n_{\text{R}}}$

4:    :

5:    $\text{G}{\}}_{n_{\text{T}}}\to {\text{H}}_{n_{\text{T}}}=\left[{\text{h}}_{n_{\text{T}}1}\cdots {\text{h}}_{n_{\text{T}}n_{\text{R}}}\right]\in C^{1\;\times \;n_{\text{R}}}$

6: **Step 1:**

7:    $\sigma =svd\left[\begin{array}{c} {\text{G}}_{\text{i}}\\ {\text{G}}_{\text{j}} \end{array}\right]$ ⇒ $diag\left(\sigma \right)=\sigma_{1},\sigma_{2},\cdots ,\sigma_{\text{r}}$

8: where

9:    $\sigma_{1}>\sigma_{2}>\cdots >\sigma_{\text{r}}$

10: **Step 2:**

11: **Calculate** **η** **between every pairs of groups**

12:    $\eta_{\text{i},\text{j}}=\frac{\sigma_{1}^{2}}{\sum_{i=1}^{r}\sigma_{\text{i}}^{2}}$

13: **Step 3:**

14:    **Find** $\Phi =\max\left(\eta_{\text{i},\text{j}}\right)$

15: Merge the groups ${\text{G}}_{\text{i}}$ and ${\text{G}}_{\text{j}}$

16: Φ > Threshold value (α)

17:    ${\text{G}}_{\text{m}}=\left[\begin{array}{c} {\text{G}}_{\text{i}}\\ {\text{G}}_{\text{j}} \end{array}\right]\in C^{\text{m}\;\times \;n_{\text{R}}}$

18: **Repeat Steps 1 and 2 until required groups remain**

## 5 Conclusion and future work

This work presents a digital beamforming (DBF) technique that significantly reduces RF hardware complexity. The proposed method is cost-effective, as it substantially lowers the number of required high-power RF chains and expensive digital-to-analog converters (DACs). Simulation results demonstrate that, with only eight RF chains and corresponding DACs, the achievable capacity closely approximates that of a conventional full-RF-complexity DBF system. The proposed architecture achieves this reduction by utilizing high-speed antenna switching within each subarray, effectively minimizing RF hardware demands. This design is particularly well-suited for large-scale multiple-input multiple-output (MIMO) systems operating in the millimeter-wave (mmWave) band, where equipping every antenna element with a dedicated RF chain is both impractical and cost-prohibitive. By grouping antennas into subarrays and applying time-division multiplexing (TDM) for switching, the system maintains high spectral efficiency while reducing overall complexity. This study highlights the importance of developing hardware capable of supporting rapid antenna switching to further enhance RF efficiency. The scenarios discussed in this paper helps to do the statistical analysis to find the actual required number of RF chains and the number of antennas in each group. It may help the designers to find the actual number of DACs and the RF chains to design low RF complexity DBF architectures for future wireless systems. Future investigations will focus on evaluating the proposed DBF architecture from a bit error rate (BER) perspective. It is anticipated that the BER performance will be favorable due to the diversity gain achieved by transmitting identical data streams across multiple antenna elements.
